# Length-based separation of *Bacillus subtilis* bacterial populations by viscoelastic microfluidics

**DOI:** 10.1038/s41378-021-00333-3

**Published:** 2022-01-19

**Authors:** Ping Liu, Hangrui Liu, Lucie Semenec, Dan Yuan, Sheng Yan, Amy K. Cain, Ming Li

**Affiliations:** 1 Suqian University, Suqian, 223800 China; 2 School of Engineering, Macquarie University, Sydney, NSW 2109 Australia; 3 Department of Physics and Astronomy, Macquarie University, Sydney, NSW 2109 Australia; 4 ARC Centre of Excellence in Synthetic Biology, Department of Molecular Science, Macquarie University, Sydney, NSW 2109 Australia; 5 Centre for Regional and Rural Futures, Deakin University, Geelong, VIC 3216 Australia; 6 Institute for Advanced Study, Shenzhen University, Shenzhen, 518060 China; 7 Biomolecular Discovery Research Centre, Macquarie University, Sydney, NSW 2109 Australia

**Keywords:** Engineering, Chemistry

## Abstract

In this study, we demonstrated the label-free continuous separation and enrichment of *Bacillus subtilis* populations based on length using viscoelastic microfluidics. *B. subtilis*, a gram-positive, rod-shaped bacterium, has been widely used as a model organism and an industrial workhorse. *B. subtilis* can be arranged in different morphological forms, such as single rods, chains, and clumps, which reflect differences in cell types, phases of growth, genetic variation, and changing environmental factors. The ability to prepare *B. subtilis* populations with a uniform length is important for basic biological studies and efficient industrial applications. Here, we systematically investigated how flow rate ratio, poly(ethylene oxide) (PEO) concentration, and channel length affected the length-based separation of *B. subtilis* cells. The lateral positions of *B. subtilis* cells with varying morphologies in a straight rectangular microchannel were found to be dependent on cell length under the co-flow of viscoelastic and Newtonian fluids. Finally, we evaluated the ability of the viscoelastic microfluidic device to separate the two groups of *B. subtilis* cells by length (i.e., 1–5 μm and >5 μm) in terms of extraction purity (EP), extraction yield (EY), and enrichment factor (EF) and confirmed that the device could separate heterogeneous populations of bacteria using elasto-inertial effects.

## Introduction

*Bacillus subtilis* is a model gram-positive, rod-shaped bacterium commonly found in soil and the gastrointestinal (GI) tract of ruminants and humans. It is generally recognized as safe (GRAS) by the US Food and Drug Administration (FDA) and widely used as a cell factory for the industrial production of heterologous proteins, pharmaceuticals, and functional biomaterials due to its propensity for genetic manipulation and ease of culture^1^. Given these favorable attributes, *B. subtilis* has served as a model organism for diverse studies on physiology, metabolism, and genetic manipulation^2^. *B. subtilis* has been intensively studied over the past decades due to its importance in the biotechnology industry and basic biological research^3^.

*B. subtilis* populations typically exhibit numerous cellular arrangements, such as single rods, chains of varying length connected by uncleaved septal wall material, sporulating cells, and biofilms comprised of matrix-producing cells. These heterogeneous cell states depend on various factors, such as the individual cell properties, the phase of growth, genetic variations, and environmental stressors^4–6^. Standard laboratory strains of *B. subtilis* are known to exist in two main morphologically distinct forms, and this cell population heterogeneity is related to the growth phase. Single motile cells or doublets occur more often during stationary phase transition, whereas long chains of sessile cells are more common in the exponential phase^7,8^. *B. subtilis* can alter cellular length via intrinsic genetic modifications, and mutant strains show distinct morphological traits^7^. Two key genes, *mreB,* and *mbl*, have been shown to play leading roles in *B. subtilis* cell shape determination^9^. Moreover, the length of *B. subtilis* can vary in response to environmental stimuli, such as temperature, antibiotic exposure, pH, and nutrient composition^10–12^.

Given the morphological and regulatory heterogeneity of *B. subtilis* cell cultures throughout the different growth phases and in response to certain conditions, it is advantageous to these fix cells after treatment at a predetermined time point for many experiments (e.g., those that assess the effects of exposure to environmental stimuli or stress). The fixation ensures that the cells maintain a homogenous morphological state and uniform growth phase during subsequent molecular and morphological examinations. Fixed cells after morphology-based separation can be visualized by microscopy, atomic force microscopy (AFM), or scanning electron microscopy (SEM) to assess changes to their ultrastructures under certain conditions, although selection and specific treatment of cells must be performed prior to fixation. Visualizing changes to bacterial cell morphology provides insight into their behaviors and mechanisms for dealing with environmental stresses, such as their response to antibiotics or host immune cells, that allow them to compete in microbial communities^13^. Furthermore, the fixed populations sorted by length can be investigated on a molecular level through DNA, RNA, and protein extraction followed by genomic, transcriptomic, or proteomic analyses to identify key molecular differences between morphologically distinct populations.

Unfortunately, traditional enrichment and separation approaches, such as centrifugal elutriation and membrane filtration, separate cells mainly by size, and they require specialized and costly equipment that also needs a substantial input size, which is not always possible with precious clinical samples. A previous technique incorporated centrifugal elutriation into a microfluidic device^14^ to address these issues and was able to separate polymer particles 1, 3, and 5 µm in diameter; however, this system has not been used for the separation of bacterial cells. Although cells with different morphologies can be isolated using micropipettes under a light microscope^15^, the process has a low throughput and is labor-intensive.

Several microfluidic techniques featuring low cost, high throughput, and high accuracy have been developed for shape-based particle and cell separation^16^. Some techniques are based on integration with externally applied fields, such as dielectrophoresis (DEP)^17^ and magnetophoresis^18^, but these always require a complex fabrication process and a bulky external setup, and external actuation may cause adverse effects on the cells (e.g., cell damage by Joule heating). Other techniques rely on internal channels or microstructure-induced microflows, such as deterministic lateral displacement (DLD)^19^, hydrophoresis^20^, and inertial focusing^21,22^. For example, DLD has been used for the separation of *Streptococcus pneumoniae* based on chain length^23^ and shape-based separation of other different bacterial species^24^ and inertial microfluidics has enabled the separation of spherical and elliptical bacterial cells^25^. However, the first two techniques require complex networks of microstructures (e.g., branched channels or post and groove arrays). Inertial microfluidics has been extensively studied for relatively large cells (typically > 2 μm), such as blood cells and circulating tumor cells (CTCs)^26–28^. Although the manipulation of bacteria with sub-micro scale resolution has been achieved recently using inertial microfluidics, this requires either a long curved microchannel^29^ or an oscillatory technique with the aid of external instruments (including pressure sources, signal generators, circuits, and valves)^27^. There have been no reports on the separation of a single species of bacterial cells using inertial microfluidics.

In this study, we adopted a viscoelastic microfluidics technique for the label-free continuous separation and enrichment of *B. subtilis* populations based on length. Although a co-flow of viscoelastic and Newtonian fluids can be used to separate both synthetic and biological particles^28,30,31^, such as the isolation of bacteria, platelets, and CTCs from whole blood^32–35^, the separation of *Staphylococcus aureus* from platelets^36^ and the separation of exosomes from other extracellular vesicles^37^, these achievements have been accomplished for two or more species and are mainly based on size. Yuan et al. recently demonstrated the shape-based separation of gram-negative *Cyanobacterial anabaena* cells using viscoelastic microfluidics^38^. Since *C. anabaena* bacterial cells are relatively large (up to ~1000 µm in length), they are easy to manipulate by microfluidic techniques. To date, it has not been shown whether elasto-inertial effects can be used to separate gram-positive bacterial cells based on shape in a co-flow system. Here, we examined the effects of different factors, including the flow rate ratio of the sheath and sample fluids, poly(ethylene oxide) (PEO) concentration, and channel length, on the lateral positions of *B. subtilis* cells of different lengths. Shorter and longer cells were found to exit mostly from the outlets closer to the channel sidewalls and center, respectively. To the best of our knowledge, this is the first report on the chain length-based separation and enrichment of a single species of gram-positive bacterial cells using viscoelastic microfluidics.

## Theory

The dynamics of fluid and particle motion in viscoelastic flows are characterized by the following three dimensionless numbers: the Reynolds number, the Weissenberg number, and the elasticity number. The values of these three dimensionless numbers at different flow rates and PEO concentrations used in the experiment are listed in the Supplementary Information (see Tables S1 and S2).

The Reynolds number, *Re*, is defined as the ratio between inertial force and viscous force^39^:

1 $$Re = \frac{{\rho UD_h}}{\eta } = \frac{{2\rho Q}}{{\eta (w + h)}}$$

where *ρ* is the fluid density, *U* is the average velocity, $D_h = 2wh/(w + h)$ is the hydraulic diameter of a rectangular channel (*w* and *h* are the channel width and height, respectively), and $Q = Q_s + Q_{sh}$ is the volumetric flow rate (*Q*_*s*_ and *Q*_*sh*_ are the sample and sheath flow rate, respectively).

The Weissenberg number, *w*_*i*_, measures the fluid elasticity effects, and is defined as^40^:

2 $$W_i = \frac{\lambda }{{t_f}} = \lambda \dot \gamma = \lambda \frac{{2U}}{w} = \frac{{2\lambda Q}}{{hw^2}}$$

where *t*_*f*_ is the characteristic time, which is approximately equal to the inverse of the average shear rate $\dot \gamma$ ($= \frac{{2U}}{w}$ or $\frac{{2Q}}{{hw^2}}$ in a rectangular microchannel).

The elasticity number, *El*, is defined as the ratio of *w*_*i*_ to *Re*:

3 $$El = \frac{{W_i}}{{Re}} = \frac{{\lambda \eta (w + h)}}{{\rho w^2h}}$$

Details of the mechanisms of particle motion dynamics under elasto-inertial effects can be found in our previous study^26^. In general, a particle flowing through a rectangular microchannel in a viscoelastic fluid experiences two main forces, elastic and inertial lift forces. The elastic lift force, *F*_*el*_, is generated by the nonuniform normal stress differences. Since the magnitude of the second normal stress difference, $N_2( = \tau _{yy} - \tau _{zz})$, is much smaller than that of the first normal stress difference $N_1( = \tau _{xx} - \tau _{yy})$ (where $\tau _{xx}$, $\tau _{yy}$, and $\tau _{zz}$ are the normal stresses exerted in the flow for the velocity gradient and vorticity direction, respectively)^41^, the effects of *N*_2_ can be neglected in dilute PEO solutions. Hence, the elastic lift force, *F*_*el*_, is proportional to the variation of *N*_1_ over the size of the particle and can be expressed as:

4 $$F_{el} = C_{el}a^3\nabla N_1 = C_{el}a^3\left( {\nabla \tau _{xx} - \nabla \tau _{yy}} \right) = - 2C_{el}a^3\eta _p\lambda \nabla _\gamma ^{ \bullet 2}$$

where C_el_ is the nondimensional elastic lift coefficient, a is the equivalent spherical diameter of the particle, and $\dot \gamma$ is the average fluid shear rate in the channel width direction.

The inertial lift force, *F*_*il*_, is composed of a wall-induced lift force, $F_{il\_w}$, that pushes the particle away from the wall, and a shear gradient-induced lift force, $F_{il\_s}$, which directs the particle from the channel center toward the walls. The net inertial lift force, *F*_*il*_, exerted on a spherical particle is given by:

5 $$F_{il} = F_{il\_w} + F_{il\_s} = C_{il}\rho U^2a^4/h^2$$

where *C*_*il*_ is the lift coefficient as a function of the Reynolds number and the normalized cross-sectional position.

As the elastic lift force *F*_*el*_ and the inertial lift force *F*_*il*_ scale differently with equivalent spherical particle diameters, particles with different morphologies (e.g., size and shape) can be directed to distinct equilibrium positions in the channel width direction under the combined effects of *F*_*el*_ and *F*_*il*_. Key external parameters that affect particle motion include the flow rate, channel dimensions, and effective relaxation time (PEO concentration). The co-flow of Newtonian and viscoelastic fluidics used in our study generates a stable interface between the sample and sheath flows close to the sidewall of the microchannel. This leads to wall-directed interfacial elastic lift forces that compete with center-directed inertial lift forces and influence the migration of cells from the Newtonian fluid to the viscoelastic fluid. It has been reported that the elastic lift force acting on the particles is dramatically altered at the interface due to the absence of elastic stresses at the Newtonian sample flow. For example, the elastic lift forces are directed toward the sidewall and centreline, respectively, when the far wall side of the particle is at the interface and the particle is fully immersed in the viscoelastic fluid^36^. A more detailed analysis of the interfacial elastic force acting on the particle in a co-flow system can be found elsewhere^36^.

## Results and discussion

**Fig. 1:**
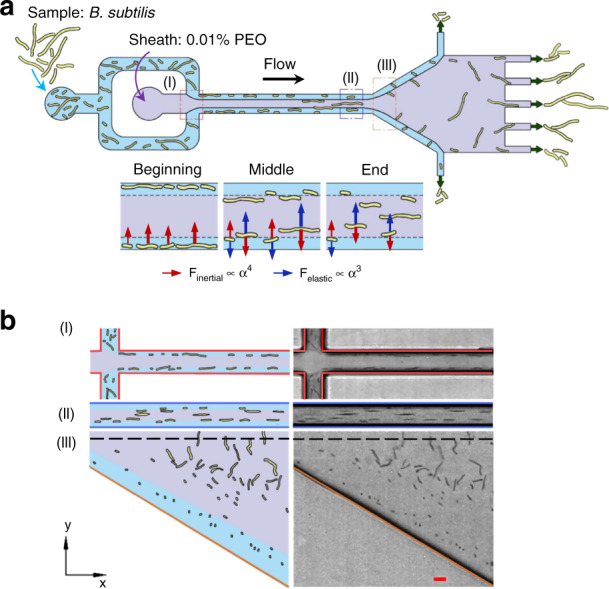
Label-free and continuous separation of *B. subtilis* by length using viscoelastic microfluidics. **a** A schematic of the viscoelastic microfluidic channel and the principles of *B. subtilis* cell separation by length (not to scale). The microchannel consists of two inlets for injecting cell suspensions and sheath fluid containing poly(ethylene oxide), a straight rectangular microchannel, an expansion region, and seven outlets. **b** A top view of the schematics (left) and experimental images (right) of the lateral position distributions of *B. subtilis* cells with different morphologies at the inlet, end of the rectangular microchannel, and expansion region. Scale bar represents 10 µm

The viscoelastic microfluidic device for the length-based separation of *B. subtilis* cells used in our experiment has four main elements: two inlets, a straight rectangular microchannel (height/width = 2.5), an expansion region, and seven outlets (see Fig. 1a). *B. subtilis* cell suspensions were injected via the side inlet, while sheath (viscoelastic non-Newtonian) fluids containing PEO were injected via the middle inlet. Due to the sheath flows, *B. subtilis* cells were prealigned into narrow focal streams along the channel sidewalls at the inlet. An interface of viscoelastic and Newtonian fluids formed close to the sidewall of the straight microchannel (~1.7 µm, see Supplementary Information Fig. S1). In the rectangular microchannel, *B. subtilis* cells with different morphologies gradually migrated toward the channel center at a length-dependent speed. Longer *B. subtilis* cells penetrated the interface and thus migrated to the channel center faster than the shorter *B. subtilis* cells that were intercepted by the interface, resulting in different lateral positions of *B. subtilis* cells that were dependent on cell length. The differences in cell length-dependent lateral positions were enhanced by the expansion region, and *B. subtilis* cells with different lengths were finally directed by different outlets: shorter and longer cells exited from the outlets close to the channel sidewalls and channel centerline, respectively. Notably, cell curvature was not found to significantly affect the lateral positions of the cells (see Fig. S2). Schematics and experimental images of the distribution of *B. subtilis* cells with different lengths at the inlet, end of the rectangular microchannel (14.78 mm downstream of the inlet), and expansion region (15.18 mm downstream of the inlet; channel width of ~235 µm) are presented in Fig. 1b, demonstrating length-based separation of *B. subtilis* cells in the proposed viscoelastic microfluidic device.

### Lateral positions depend on *B. subtilis* cell length

**Fig. 2:**
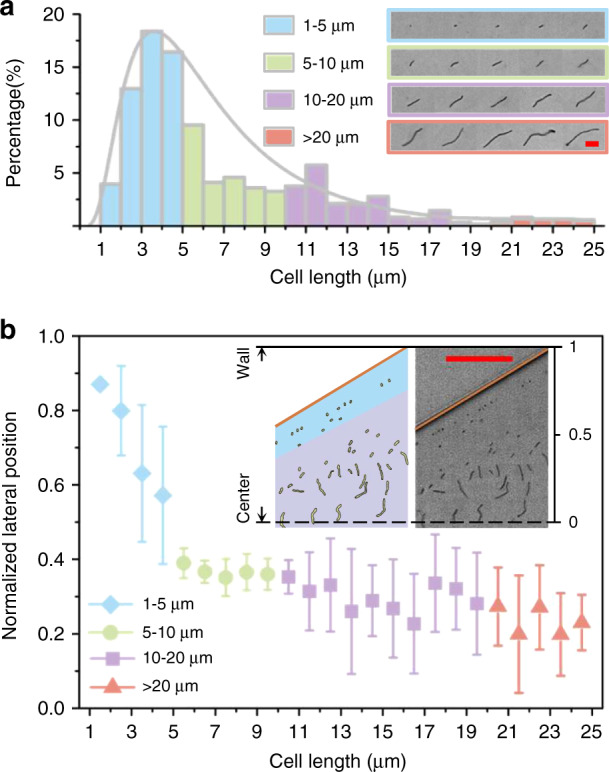
The lateral positions of *B. subtilis* cells depend on cell length. **a** Histogram of the chain length distribution of four groups of *B. subtilis* cells: 1–5 μm (blue), 5–10 μm (green), 10–20 μm (purple), and >20 μm (pink). The inserts are images showing four groups of *B. subtilis* cells with different lengths. The scale bar represents 10 µm. **b** Plot of the normalized lateral positions of the four groups of *B. subtilis* cells with different lengths. The error bars indicate the standard deviations obtained from at least 20 measurements. The insets are a schematic (left) and an experimental image (right) of the normalized lateral position distributions of *B. subtilis* cells of different lengths at the expansion region. The scale bar represents 50 µm

We examined the lateral positions of *B. subtilis* with different morphologies, which were found to be dependent on cell length. The *B. subtilis* cell suspension and 100 ppm PEO solution were injected into the viscoelastic microfluidic device at flow rates of 5 and 40 μL/min, respectively. The corresponding Re value was 24.91 and Wi was 9.23. These two dimensionless parameters were calculated using Eqs. (1) and (2), with a channel width of 20 μm and a height of 50 μm and the values of density ρ, dynamic viscosity η and effective relaxation time λ of the PEO solutions can be found in Table S3. The lengths of the *B. subtilis* cells ranged from 1.2 to 24.6 μm with an approximate medium value of 6.8 ± 0.3 μm, whereas the widths of the *B. subtilis* cells were all approximately 0.8 μm (see Fig. 2a). We divided the *B. subtilis* cells into four groups based on cell length: 1–5 μm, 5–10 μm, 10–20 μm, and >20 μm, that represent 51.4%, 24.9%, 20.5%, and 3.2% of the population, respectively. Fig 2b compares the normalized lateral positions of the four groups of *B. subtilis* cells at an expansion region (15.2 mm downstream of the inlet, channel width of ∼260 μm). An expansion region rather than a 20 μm wide rectangular channel region was selected for measurements to easily visualize the differences in cell lateral positions. Since two sets of lateral positions symmetric to the channel centerline were found for *B. subtilis* cells with different lengths, only the cell lateral positions in half of the expansion region were shown (see Fig. 2b, insets). The results showed that the normalized lateral position generally decreases with increasing *B. subtilis* cell length; longer cells were present in lateral positions closer to the channel centerline than shorter cells (see Fig. 2b and Supplementary Movie S1).

Moreover, we investigated the capability of inertial microfluidics alone for the length-based separation of *B. subtilis* cells using the same device (see Supplementary Information Figure S3). The lateral positions of the longer cells were found to be closer to the channel wall, reducing the difference in lateral position for the two groups of cells (i.e., cells <5 µm and cells >5 µm). This finding was confirmed after comparing the lateral positions of *B. subtilis* cells at the outlets (see Supplementary Information Fig. S2). A portion of longer cells (> 5 µm) and shorter cells (<5 µm) were found to exit from the same outlet (see Fig. S4a) using inertial microfluidics alone; hence, reduced separation efficiency of these two groups based on chain length occurred. We also tested the ability of the device to separate *B. subtilis* cells of various lengths using viscoelastic microfluidics alone (see Figure S5), and no clear separation was observed due to the large overlaps in the cell lateral positions.

### Effects of the flow rate ratio

We first investigated the effects of the flow rate ratio *α* ( = *Q*_*sh*_/*Q*_*s*_) on the lateral positions of *B. subtilis* cells with different morphologies. The flow rate of the sheath fluid containing 100 ppm PEO in PBS was fixed at 40 μL/min, while the sample fluid containing *B. subtilis* cells of various lengths was injected into a 15 mm long microchannel at five different flow rates, 2, 3, 4, 5, and 10 µL/min. The flow rate ratios *α* were 20, 13.3, 10, 8, and 4 respectively. The corresponding Re, Wi, and El values are listed in Supplementary Information Table S1. According to Eqs. (1) and (2), both Re and Wi increased with an increase in the total flow rate (also a decrease in *α*), which leads to enhanced inertial and elastic effects. However, El remained constant at 0.37 (see Eq. (3)), indicating that inertial lift force dominates in the entire range of *α*^28^.

**Fig. 3:**
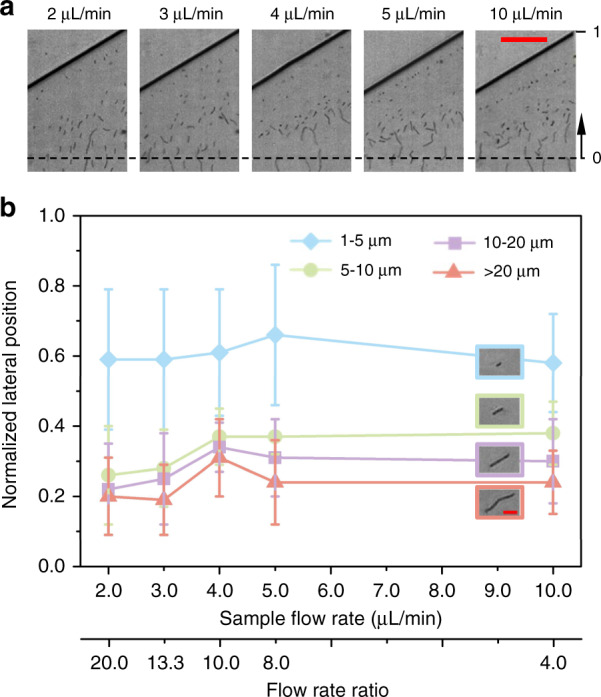
Effects of flow rate ratio on the separation of *B. subtilis* cells using a 100 ppm PEO solution in a 15 mm long rectangular microchannel. The sheath flow rate was fixed at 40 µL/min, while the sample fluid varied, at 2, 3, 4, 5, and 10 µL/min. The Re values were 23.25, 23.8, 24.35, 24.91, 27.67, respectively, and Wi values were 8.61, 8.82, 9.02, 9.23, 10.25, respectively. **a** Experimental images of *B. subtilis* cells with various lengths at the expansion region for the five different sample flow rates. The black dashed lines represent the channel centerlines. The scale bar represents 50 µm. **b** Plots of the average normalized lateral positions for the four groups of *B. subtilis* cells with different lengths: 1–5 μm (blue), 5–10 μm (green), 10–20 μm (purple), and >20 μm (pink). The error bars indicate the standard deviations obtained from at least 100 measurements

When *α* decreased from 20 (*Q*_*s*_ = 2 µL/min) to 10 (*Q*_*s*_ = 4 µL/min), the lateral positions of the four groups of *B. subtilis* cells were found to migrate slightly toward the channel wall (see Fig. 3). This is mainly a result of the slightly increased dominant inertial lift forces, which direct the long cells that traverse the interface and are located in the viscoelastic non-Newtonian fluid toward the channel sidewall. As *α* was further decreased to 8 (*Q*_*s*_ = 5 µL/min), the three groups of longer *B. subtilis* cells (i.e., 5–10 μm, 10–20 μm, and >20 μm) appeared to migrate slightly toward the channel centerline, while the shorter *B. subtilis* cells (i.e., 1–5 μm) appeared to migrate slightly toward the channel sidewall. This resulted in an increase in the gap between the average normalized lateral positions of the four groups of *B. subtilis* cells (see Fig. 3b). This result is probably because the focusing of the cells at the beginning of the straight channel decreased, reducing the cell-to-cell interactions in the regions closer to the channel wall. Upon decreasing *α* further to 4 (*Q*_*s*_ = 10 µL/min), shorter *B. subtilis* cells (i.e., 1–5 μm) tended to move closer to the channel centerline, reducing the gap between the average normalized lateral positions. This is mainly because in Newtonian fluids, the increasing inertial lift forces direct shorter *B. subtilis* cells away from the wall.

We also examined how the sheath flow rate affected the migration of *B. subtilis* cells with different morphologies by increasing the sheath flow rate from 10 to 50 µL/min while keeping the sample flow rate constant at 5 µL/min (see Supplementary Information Figure S6). The results showed that there was no significant variation in the average normalized lateral positions of *B. subtilis* cells at different sheath flow rates (except when the shorter *B. subtilis* cells with lengths of 1–5 μm appeared to migrate toward the channel wall when the sheath flow rate was 40 µL/min). The length-based separation of *B. subtilis* cells was found to not be very sensitive to flow rates, probably because both the elastic and inertial lift forces are proportional to *U*^2^ (see Eqs. (4) and (5)). This observation agrees with the previous study by Lu et al.^42^, where the lateral positions of spherical and peanut particles remained almost unchanged at different flow rates.

### Effects of PEO concentration

We further investigated the effects of PEO concentration *c* on the lateral positions of *B. subtilis* cells with different morphologies. Here, we used three different concentrations of PEO solutions, 100, 500, and 1000 ppm. The sample and sheath flows were injected into the microchannel with a length of 15 mm at the fixed flow rates of 5 μL/min and 40 μL/min, respectively. The Re values were 24.91, 21.52, and 18.39, respectively, and the Wi values were 9.23, 26.25, and 41.25, respectively (see Table S2).

**Fig. 4:**
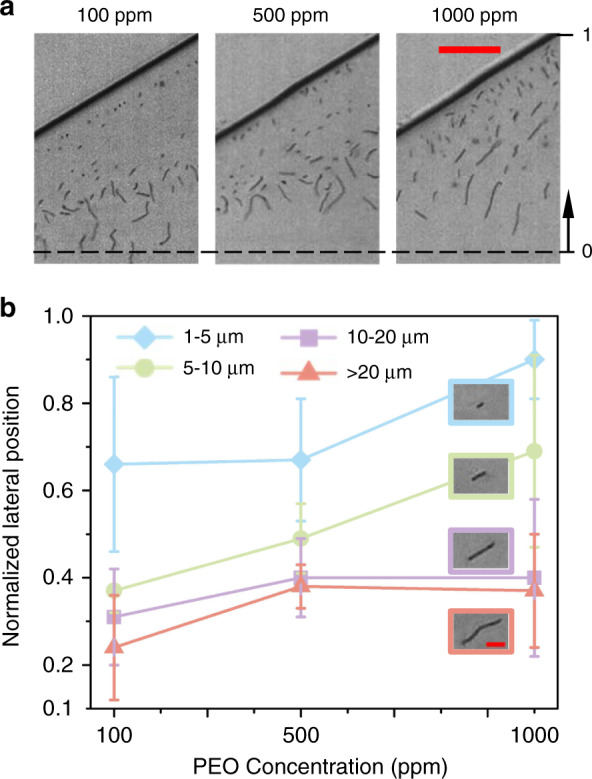
Effects of PEO concentration on the separation of *B*. *subtilis* cells in a 15 mm long rectangular microchannel with three different concentrations of PEO solutions: 100, 500, and 1000 ppm. The flow rates of the sample and sheath were 5 µL/min and 40 µL/min, respectively. The Re values were 24.91, 21.52, 18.39, respectively, and the Wi values were 9.23, 26.25, 41.25, respectively. **a** Experimental images of *B. subtilis* cells of various lengths at the expansion region for the three different PEO concentrations. The black dashed lines represent the channel centerlines. The scale bar represents 50 µm. **b** Plots of the average normalized lateral positions for four groups of *B. subtilis* cells with different lengths: 1–5 μm (blue), 5–10 μm (green), 10–20 μm (purple), and >20 μm (pink). The error bars indicate the standard deviations obtained from at least 100 measurements

When the PEO concentration *c* was 100 ppm (El = 0.37), there was a clear difference in the lateral positions of the four groups of *B. subtilis* cells (see Fig. 4). A portion of short *B. subtilis* cells (i.e., 1–5 μm) were trapped at the interface of the viscoelastic and Newtonian fluids, while longer *B. subtilis* cells penetrated the interface and reached the lateral equilibrium positions in the viscoelastic fluid that were dependent on cell length. When *c* increased to 500 ppm (El = 1.22), the three groups of longer *B. subtilis* cells (i.e., 5–10 μm, 10–20 μm, and >20 μm) tended to move closer to the channel wall, resulting in a decrease in the distance between the normalized lateral positions. This is because wall-directed elastic lift forces began to dominate the center-directed inertial lift force at the interface, and a longer cell length is required to penetrate the interface. Tian et al.^36^ observed that larger particles (i.e., 2 µm in diameter) migrated from the centerline to the sidewalls when the PEO concentration increased from 100 ppm to 1000 ppm.

As *c* was further increased to 1000 ppm (EI = 2.24), the elastic lift force became more dominant. A larger portion of short *B. subtilis* cells in the first two groups (i.e., 1–5 μm and 5–10 μm) were trapped at the interference, resulting in a shift in the average normalized lateral position of these two groups toward the channel sidewall. However, the average normalized lateral position of *B. subtilis* cells in the other two groups (i.e., 10–20 μm and >20 μm) shifted slightly toward the microchannel centerline. The PEO concentration *c* of the sheath fluid has complex effects on cell migration^37^. As the effective relaxation time λ of the PEO concentration increases with *c*^1.65^, at a higher *c*, Wi is larger (see Eq. (2)), and the wall-directed interfacial elastic lift force is more dominant (El is larger, see Eq. (3)), resulting in more cells trapped at the interface. For longer cells that are located fully in the viscoelastic fluid, the applied elastic lift forces them toward the channel centerline. We noted that the separation performance is more sensitive to PEO concentrations than flow rates. These results agree with previous findings by Tian et al.^36^, in which a co-flow of viscoelastic and Newtonian fluids was used to separate 1 and 2 μm particles.

It is worth noting that we also tested the separation performance of the device at a relatively low PEO concentration (i.e., 50 ppm). We found that a portion of short *B. subtilis* cells (i.e., 1–5 μm) were directed away from the wall and that the lateral positions of the longer cells were closer to the channel wall (see Fig. S7), leading to a reduced decrease in the distance between the lateral positions of the two cell groups (i.e., < 5 µm and >5 µm) compared to the results when 100 ppm PEO was used.

### Effects of channel length

**Fig. 5:**
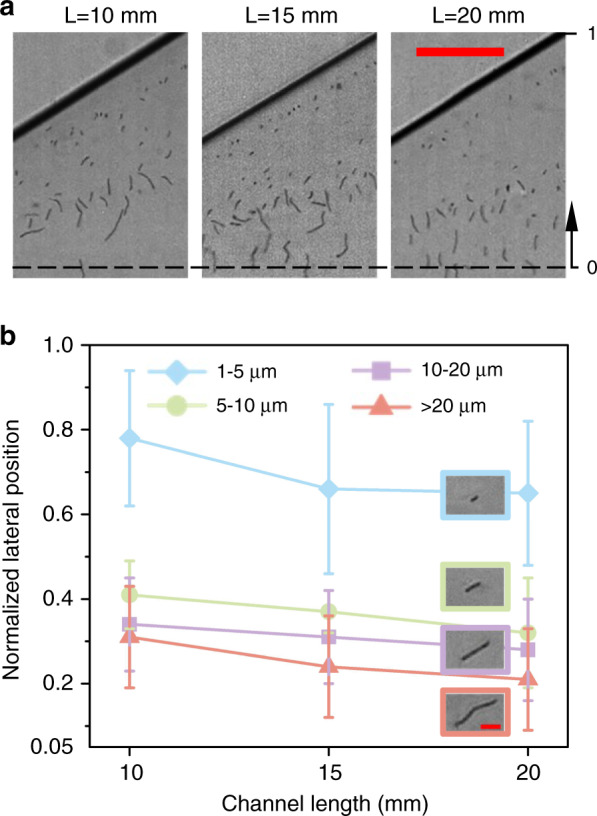
Effects of channel length on the separation of *B. subtilis* cells using a 100 ppm PEO solution within a rectangular microchannel with three different lengths, 10, 15, and 20 mm. The flow rates of the sample and sheath were 5 µL/min and 40 µL/min, respectively. The Re and Wi values were 24.91 and 9.23, respectively. **a** Experimental images of *B. subtilis* cells with different lengths at the expansion region of the channels with three different lengths. The black dashed lines represent the channel centerlines. The scale bar represents 50 µm. **b** Plots of the average normalized lateral positions for the four groups of *B. subtilis* cells with different lengths: 1–5 μm (blue), 5–10 μm (green), 10–20 μm (purple), and >20 μm (pink). The error bars indicate the standard deviations obtained from at least 100 measurements

We also investigated the effects of channel length on the lateral positions of *B. subtilis* cells with different morphologies. Here, we used the microchannels with three different lengths, 10, 15, and 20 mm, while the width and height of the channel were fixed at 20 and 50 μm, respectively. The sample flow and sheath flow (containing 100 ppm PEO in PBS) were fixed at the flow rates of 5 μL/min and 40 μL/min, respectively. The corresponding Re was 24.91, Wi was 9.23, and El was 0.37. Figure 5 compares the normalized lateral positions of the four groups of *B. subtilis* cells at the expansion region (i.e., channel width of ∼260 μm, 200 μm downstream of the end of the straight channel). The results showed that longer *B. subtilis* cells had lateral positions closer to the channel centerline than shorter *B. subtilis* cells in all three channels of different lengths. This is because of the cell-length-dependent penetration of the interface between the viscoelastic and Newtonian fluids. Additionally, all four groups of *B. subtilis* cells migrated slightly toward the channel centerline with increasing channel length. This indicates that the combined effects of elastic and inertial lift forces on the length-dependent lateral displacement of *B. subtilis* cells are cumulative, which agrees with our previous study^26^ that cells of different sizes migrated toward the centerline when the microchannel length increased from 10 mm to 20 mm. Therefore, we need to carefully select the optimum channel length to maximize the distance between the lateral positions of the cells with different lengths.

### Length-based separation of *B. subtilis* cells at the outlets

**Fig. 6:**
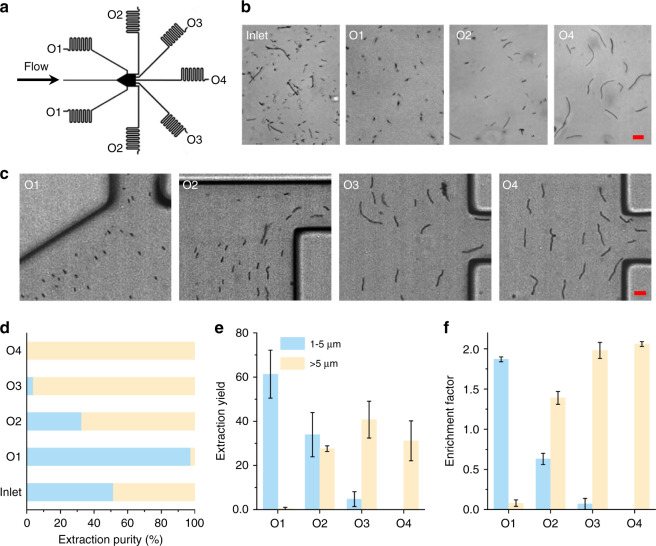
Separation and enrichment of *B. subtilis* cells with different lengths at the outlets using 100 ppm PEO solution in a 15 mm long rectangular microchannel. The flow rates of sample and sheath fluids were 5 µL/min and 40 µL/min, respectively. **a** An illustration of the seven outlets for collecting *B. subtilis* cells of different lengths. **b** Snapshot images comparing the proportion of *B. subtilis* cells at the inlet and outlets. **c** Superimposed experimental images showing that *B. subtilis* with different lengths are more likely to exit from different outlets: short and long cells are more likely to exit from outlets closer to the sidewall (O1) and centerline (O4), respectively. Scale bar represents 10 µm. **d** Comparison of the EP for the two groups of *B. subtilis* cells: 1–5 μm (blue) and >5 μm (yellow) at the inlet and each outlet. **e**, **f** Bar graphs of (**e**) EY and (**f**) EF for the two groups of *B. subtilis* cells with different lengths for each outlet. The error bars represent the standard deviations of three measurements

Based on the differences in the lateral positions of *B. subtilis* cells that are dependent on cell length, we achieved the label-free, continuous separation of *B. subtilis* cells by length at the outlets. We injected cell-loaded sample fluid and sheath fluid (containing 100 ppm PEO in PBS) into a 15 mm long microchannel at flow rates of 5 μL/min and 40 μL/min, respectively (Re = 24.91, Wi = 9.23, El = 0.37). We designed a serpentine microchannel as a fluidic resistor for each outlet (see Fig. 6a), aiming to reduce the flow rate distortion caused by any small variation in the fluidic resistance at the outlets (e.g., tubing length variation and clogging from debris)^21^. *B. subtilis* cells with different lengths (i.e., in the range of 1.2–24.6 μm) at the inlets were able to migrate toward and be collected at the different outlets (see Fig. 6b, c).

Shorter *B. subtilis* cells were found to mainly exit through side outlet 1, while longer *B. subtilis* cells were mainly collected from middle outlet 4 at the channel centerline. This length-based separation of *B. subtilis* cells with different lengths at the outlets can also be visualized in Supplementary Movies S2 and S3.

To quantify the efficiency of the length-based separation of *B. subtilis* cells, we used three parameters^26^: extraction purity (EP), extraction yield (EY), and enrichment factor (EF) (see Fig. 6d-f). Shorter *B. subtilis* cells with a length of 1–5 μm (blue) were mainly collected from outlet 1 (O1), with an EP of 97.5% (Fig. 6d) and an EY of 61.3% (Fig. 6e). The EP of shorter *B. subtilis* cells at O3 was 3.8%. This presence of shorter *B. subtilis* cells at O3 might be caused by cell adhesion during sample injection and separation, cell-to-cell interactions, or experimental uncertainties. Longer *B. subtilis* cells with a length of >5 μm (yellow) were found to have relatively high EPs of 96.19% and 100% from outlet 3 (O3) and outlet 4 (O4), respectively (see Fig. 6d). This yielded EFs of 1.97 and 2.06 at O3 and O4, respectively, for *B. subtilis* cells with a length of >5 μm (see Fig. 6f). Additionally, the separation of the four groups of *B. subtilis* cells (i.e., 1–5 μm, 5–10 μm, 10–20 μm, and >20 μm) was analyzed (see Supplementary Information Fig. S8). We also examined the separation efficiency in terms of EP under different experimental conditions (see Supplementary Information Figure S9), which resulted in a lower EP for the shorter *B. subtilis* cells (i.e., 1–5 μm) at outlet 1 with higher PEO concentrations. This is because longer cells tended to move toward the channel wall when *c* was increased (see Fig. 4). Moreover, a portion of shorter *B. subtilis* cells were found to exist from outlet 4 when the sample flow rate was increased to 50 μL/min, as they appeared to migrate toward the channel center under these conditions (see Fig. S6).

Our approach allowed for the successful separation of *B. subtilis* cells into two distinct groups (cells < 5 µm and cells > 5 µm), distinguishing cells in exponential and in stationary growth phases^43^. *B. subtilis* cells have distinct sizes according to their growth phase. They are approximately 2.7 µm long during the stationary phase^43^ and significantly longer during the exponential phase, where they can form chains of filaments^44^. In addition to different phases of growth, the morphology and size variations of *B. subtilis* are also affected by the growth conditions (e.g., nutrient composition^45^ and antibiotics^46,47^) in which *B. subtilis* is cultured.

The geometry of the device and the properties of viscoelastic fluids can be optimized to improve separation efficiency and throughput. For example, specific channel designs can enhance the efficiency of cell separation, including sudden expansion^21,48^, asymmetrical arranged outlet branches and fluidic resistors^21,49,50^ and a cascade of microchannels^51^. In addition, some biological polymers, e.g., λ-DNA, hyaluronic acid, xanthan gum, and extracellular molecules, can be used as alternatives to synthetic polymers (i.e., PEO) in non-Newtonian fluids for particle manipulation^37,52^.

## Methods and materials

### Device fabrication

The design of the microfluidic device consists of four main elements: two inlets for separately introducing the viscoelastic sheath and sample fluids, a straight rectangular channel with a width of 20 μm and three different lengths (10, 15, and 20 mm), an expansion region (with an opening angle of 60°, a total length of 0.9 mm, and a maximum width of 0.6 mm), and seven outlets with customized fluidic resistors. The microchannel is uniform with a height of 50 μm. The master mold made of the negative photoresist SU-8 2050 (MicroChem, Newton, MA, USA) was patterned on a silicon substrate using a laser direct writer (MicroWriter ML3, Durham Magneto Optics, Durham, UK). Degassed poly(dimethyl siloxane) (PDMS; Sylgard 184, Dow Corning, Midland, MI, USA) in liquid form was prepared by mixing the PDMS base with the curing agent in a weight ratio of 10:1, casting over the SU-8 mold, and baking in an oven at 60 °C for at least 2 h. The PDMS slab containing a negative replica of the microchannels was then peeled off of the mold, and the openings for the inlets and outlets were punched. The PDMS slab and a glass substrate were treated with a plasma cleaner (PX 250, March Instruments, Concord, CA, USA) and assembled. To enhance bonding, the assembled microdevice was placed on a hotplate at 95 °C for 2 min.

### Viscoelastic fluid preparation

The viscoelastic sheath fluids used in the experiment, PEO solutions with three different concentrations (100, 500, and 1000 ppm), were prepared by dissolving PEO powder (*M*_w_ = 600 kDa, 182028, Sigma–Aldrich, St. Louis, MO, USA) in deionized (DI) water. To completely dissolve the PEO powder, short vortexing (~ 10 s) followed by overnight gentle shaking at a speed of 100 rpm/min using an orbital shaker (CLS6791, Corning, Corning, NY, USA) was performed. The properties of the prepared PEO solutions are listed in the Supplementary Information (see Table S3), which were calculated as mentioned in our previous work^26^. The effective relaxation times of the prepared PEO solutions *λ* were estimated from previous times measured by capillary breakup extension rheometry (CaBER)^53^. *λ* can be defined as $\lambda = 18\lambda _z(c/c^ \ast )^{0.65}$, where *c* is the polymer concentration and $c^ \ast = 0.77/[\eta ]$ is the polymer overlap concentration (the intrinsic viscosity $[\eta ] = 0.072M_W^{0.65}$)^54^_._ $\lambda _z = f[\eta ](M_w)\eta _s/RT$ is the relaxation time predicted by Zimm theory^54,55^, where *f* ( = 0.463) is the prefactor dependent on solvent quality, *η*_*s*_ ( = 1 × 10^-3^ Pa·s) is the solvent viscosity, *R* ( = 8.314 J mol^−1^K) is the gas constant, and *T* ( = 293 K) is the absolute temperature. Since the polymeric contribution to the solution viscosity *η*_*p*_ can be expressed as $\left[ \eta \right]c\eta _s = 0.072c\eta _sM_W^{0.65}$, the dynamic viscosity $\eta = \eta _s + 0.072c\eta _sM_W^{0.65}$_._

### Analysis of separation performance

The separation efficiency can be characterized by three parameters^56^. The EP considers the proportion of the number of a cell group *a* in outlet *i* relative to the total number of cells extracted from this outlet; EP reports the cell composition of a given outlet. EY is calculated as the number of cell groups *a* extracted from outlet *i* over the total number of this cell group injected from the inlet; EY is the outlet collection efficiency of a given cell group. EF is defined as the proportion of cells *a* in outlet *i* to the proportion of cells at the inlet.

$$EP = \frac{{N_a\left( {outlet_i} \right)}}{{N_{total}\left( {outlet_i} \right)}},\;EY = \frac{{N_a\left( {outlet_i} \right)}}{{N_a\left( {inlet} \right)}},$$

$$EF = \frac{{N_a(outlet_i)/N_{total}(outlet_i)}}{{N_a(inlet)/N_{total}(inlet)}}.$$

### Cell preparation

*B. subtilis* ATCC 6633 cells were grown overnight at 37 °C with shaking at 200 rpm in 5 mL of Luria-Bertani broth (51208, Sigma–Aldrich, St. Louis, MO, USA). Overnight cultures were spun down at 5000 g at 4 °C for 5 min and washed with 5 mL of ice-cold 1× phosphate-buffered saline (PBS; 10010023, Thermo Fisher Scientific, Waltham, MA). This step was repeated once for a total of two washes. To characterize the trajectories of the fixed *B. subtilis* cells in the viscoelastic microfluidic device, the washed cell suspensions were added to 4.5 mL of ice-cold 70% ethanol while vortexing gently. Cells were then incubated at 4 °C for 2 h followed by centrifugation at 1000 g for 5 min. The supernatant was carefully removed, and the cells were resuspended in 1 × PBS and incubated at ambient temperature for 15 min. These fixed cells were used for the cell separation experiment in this study, as they can resist lysis for a long time at 4 °C in a refrigerator. Then, 25 μL of methylene blue (50484, Sigma–Aldrich, St. Louis, MO, USA) was added to 500 μL of final solution with a cell concentration of 2 × 10^7^ cell/mL and cocultured for 20 min for downstream experiments.

### Experimental setup

Before the experimental operation, the microdevices were prefilled with 0.1% Tween^®^ 20 (P9416, Sigma–Aldrich, St. Louis, MO, USA) supplemented with deionized (DI) water to prevent the cells from adhering to the channel walls. Afterwards, *B. subtilis* cell suspensions (sample fluid) and PEO solution (sheath fluid) were simultaneously injected into the microchannel via two separate inlets using two syringe pumps (Fusion 100, Chemyx, Stafford, TX, USA). The trajectories of the *B. subtilis* cells were monitored and recorded with a high-speed camera (Phantom Miro LC320S, Vision Research, Wayne, NJ, USA) mounted on an inverted microscope (Eclipse Ti-U, Nikon, Tokyo, Japan) at a fixed exposure time of 30 µs. Recorded videos and images were postprocessed and analyzed by ImageJ software (National Institutes of Health (NIH), Bethesda, MD, USA). The lateral position distributions of *B. subtilis* cells were calculated based on the distance between the centroids of the cells and the channel centerline using stacked images captured 100 μm downstream of the end of the straight channel. The lengths of the cells were determined by measuring the Feret diameters of the cells. Sorted cells were collected at the outlets and counted to calculate separation efficiency in terms of EP, EY, and EF.

## Conclusion

In this work, we demonstrated the label-free continuous separation of *B. subtilis* cells by length using a co-flow of viscoelastic and Newtonian fluids in a straight rectangular microchannel. *B. subtilis* cells varying in morphology were found to have different lateral equilibrium positions, dependent on cell length due to elasto-inertial effects and separation. The results revealed that these factors generally have a greater effect on the migration of shorter cells (i.e., <5 μm) than that of longer cells (i.e., >5 μm). *B. Subtills* cell separation did not increase monotonically with PEO concentration because of the combined elastic and inertial effects, but microchannel length strongly influenced cell migration because the combined effects of elastic and inertial lift forces are cumulative. We performed a systematic experimental study of the influences of the flow rate ratio, PEO concentration, and channel length on the lateral positions of *B. subtilis* cells of different lengths. Moreover, length-based separation and enrichment of *B. subtilis* cells were achieved at the outlets. Shorter cells were found to have a high EP of 97.5% at the outlet closest to the channel wall, and longer cells were mainly collected from the outlet in the channel center with an EY of 65.2% and an EF of up to 6.88. Our study is the first to demonstrate that viscoelastic microfluidics can be adopted for the separation of bacterial cells by length, an important biomarker. To date, few theoretical models have been developed to predict particle migration in Newtonian and viscoelastic fluidics co-flow systems. However, several computational fluid dynamics simulations^28,57^ and novel imaging instruments^52,58^ (e.g., digital holography) have been adopted to investigate the velocity profiles and particle focusing patterns. These approaches can potentially facilitate detailed investigations to elucidate the underlying mechanisms. The current platform is expected to be used as a powerful tool for basic studies and industrial applications with bacterial cells by integrating functional components (e.g., focusing and imaging)^59,60^, functional genomics, and metabolic and genetic engineering techniques.

## Supplementary information

Supplemental Material 08112021

Movie S-1

Movie S-2

Movie S-3
