# Supplementary material for: Length-based separation of Bacillus subtilis bacterial populations by viscoelastic microfluidics
Source: Microsyst Nanoeng. 2022 Jan 19;8:7. doi: 10.1038/s41378-021-00333-3 (PMC8766588; doi:10.1038/s41378-021-00333-3)
Supplement: Supplementary file 1 — Supplemental Material 08112021 [file 41378_2021_333_MOESM1_ESM.docx]

Supplementary Information

**Length-based separation of *Bacillus subtilis* bacterial populations by viscoelastic microfluidics**

Ping Liu^a,b,+^, Hangrui Liu^c,+^, Lucie Semenec^d^, Dan Yuan^e^, Sheng Yan^f^, Amy K. Cain^d^, Ming Li^b,g*^

^a^ Suqian University, Suqian 223800, China

^b^ School of Engineering, Macquarie University, Sydney, NSW 2109, Australia

^c^ Department of Physics and Astronomy, Macquarie University, Sydney, NSW 2109, Australia

^d^ ARC Centre of Excellence in Synthetic Biology, Department of Molecular Science, Macquarie University, Sydney, NSW 2109, Australia

^e^ Centre for Regional and Rural Futures, Deakin University, Geelong, Victoria 3216, Australia

^f^ Shenzhen University, Shenzhen, China

^g^ Biomolecular Discovery Research Centre, Macquarie University, Sydney, NSW 2109, Australia

^+^ equal contribution

^*^ corresponding author, E-mail: [ming.li@mq.edu.au](mailto:ming.li@mq.edu.au)

**Table S1.** Dimensionless numbers at different flow rates (when c=100 ppm, El=0.37)

|  | $Q_{sh}$=40 µL/min | | | | |
| --- | --- | --- | --- | --- | --- |
| Total flow rate (µL /min) | 42 | 43 | 44 | 45 | 50 |
| Reynolds number (Re) | 23.25 | 23.8 | 24.35 | 24.91 | 27.67 |
| Weissenberg number (Wi) | 8.61 | 8.82 | 9.02 | 9.23 | 10.25 |
| Elasticity number (El) | 0.37 | | | | |

**Table S2.** Dimensionless numbers at different concentrations (when$Q_{s}$=5 µL/min, $Q_{sh}$=40 µL/min)

| PEO concentration (c,ppm) | 100 | 500 | 1000 |
| --- | --- | --- | --- |
| Reynolds number (Re) | 24.91 | 21.52 | 18.39 |
| Weissenberg number (Wi) | 9.23 | 26.25 | 41.25 |
| Elasticity number (El) | 0.37 | 1.22 | 2.24 |

**Table S3.** Properties of PEO solutions used in the experiment

| PEO concentration(c,ppm) | 100 | 500 | 1000 |
| --- | --- | --- | --- |
| Density ρ (g/cm^3^) | 1.210 | 1.210 | 1.210 |
| Dynamic viscosity η (mpa.s) | 1.041 | 1.205 | 1.41 |
| Ovelap concn c^*^ (ppm) | 1877 | 1877 | 1877 |
| Concn ratio c/c^*^ | 0.053 | 0.266 | 0.533 |
| Zimm relaxation time,λ_z_ (ms) | 0.046 | 0.046 | 0.046 |
| Effective relaxation time, λ (ms) | 0.123 | 0.35 | 0.55 |

Figure S1 Measurement of widths of sample flow (0.79 µm polystyrene beads in PBS) and sheath flow (pure PBS) at (a) the beginning of the straight channel and (b) the expansion region using 0.79 µm polystyrene beads in PBS as sample flow and pure PBS as sheath flow. The white dashed lines represent the channel walls. The sample flow rate is fixed at 5 µL/min, while sheath flow rate is at 40 µL/min. Scale bar = 50 µm


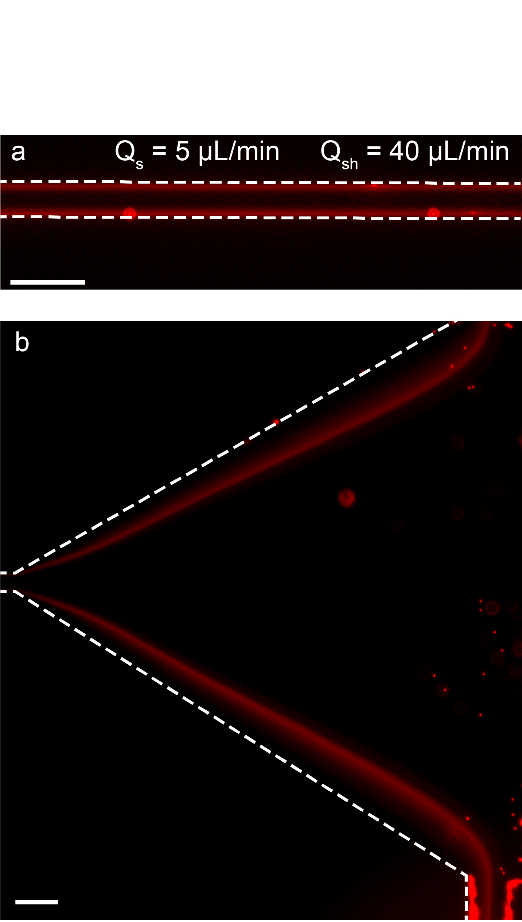

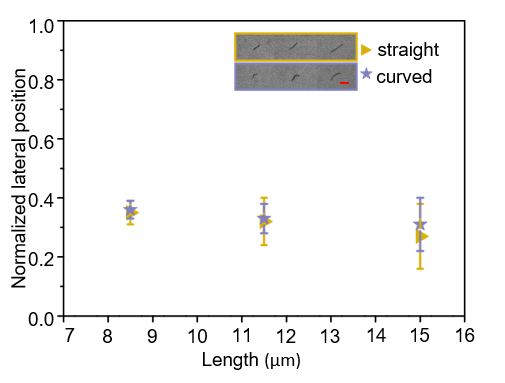


Figure S2 A plot of normalized lateral positions for straight (yellow) and curved (purple) B. subtilis cells having the same lengths. The scale bar represents 10 µm. The error bars indicating the standard deviation obtained from at least 20 measurements.


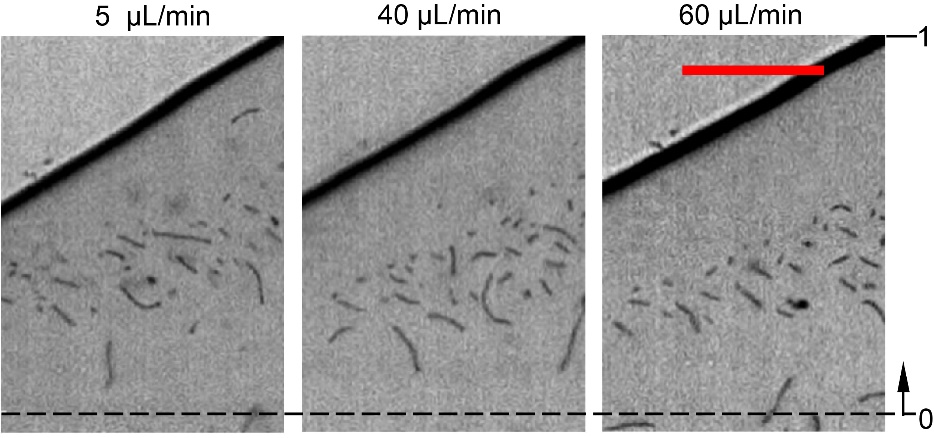


Figure S3 Comparison of lateral positions of *B. subtilis* cells with different lengths at the expansion region using inertial microfluidics alone in a 15 mm long rectangular microchannel when both sample fluid and sheath fluid are PBS. The sample flow rate is fixed at 5 μL/min, while sheath fluid has three different flow rates: 5, 40 and 60 µL/min. The black dashed lines represent channel centerlines. Scale bar represents 50 μm.


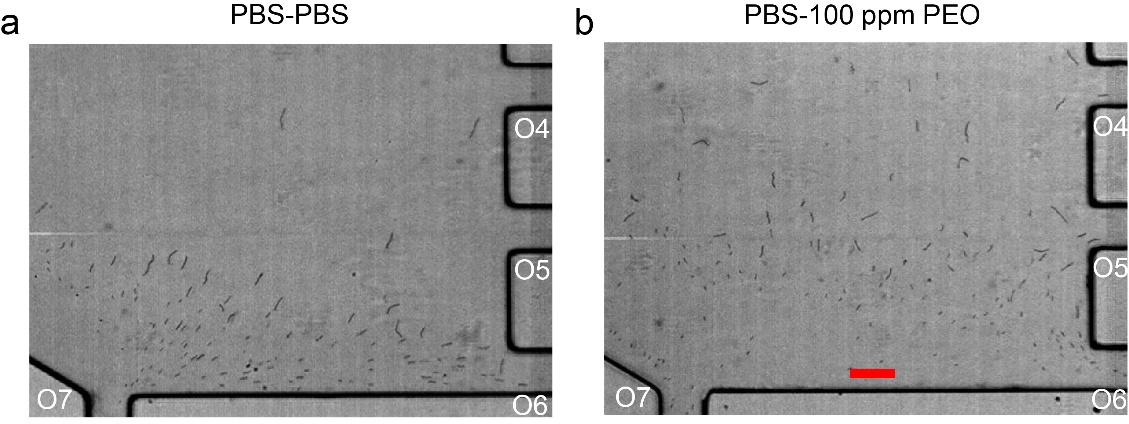


Figure S4 Comparison of lateral positions of *B. subtilis* cells with different lengths at the outlets using (a) PBS and (b) 100 ppm PEO solution in a 15 mm long rectangular microchannel. The flow rates of sample and sheath fluids are 5 µL/min and 40 µL/min, respectively, in both cases. The scale bar represents 50 µm.

Figure S5 Comparison of lateral positions of B. subtilis cells with different lengths at the expansion region using elastic-inertial fluidics and viscoelastic microfluidics alone (100 ppm PEO) in a 15 mm long rectangular microchannel, when both sheath fluid and sample fluid contain 100 ppm PEO. The sample flow rate is fixed at 5 µL/min, while sheath flow rate is 40 µL/min. The black dashed lines represent channel centerlines. Scale bar represents 50 μm.


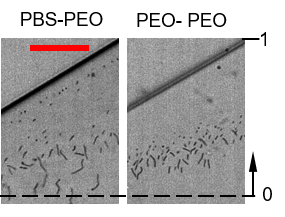


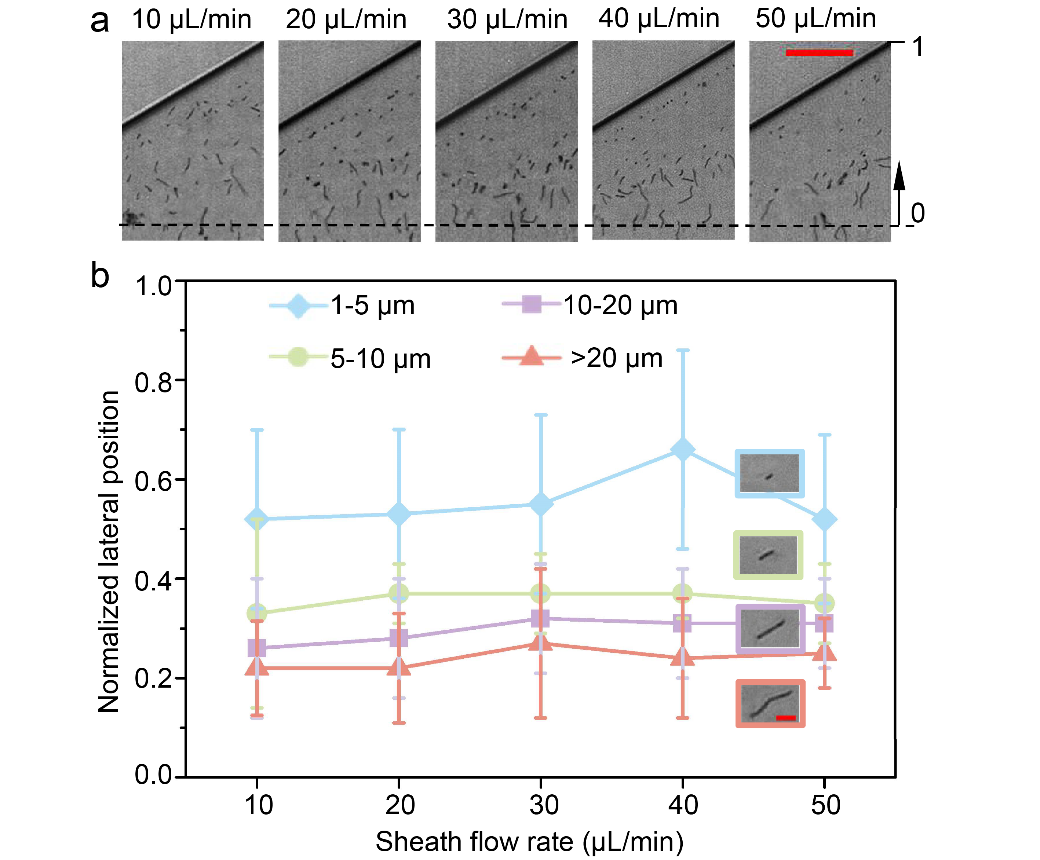


Figure S6 Effect of sheath flow rate on the separation of *B. subtilis* cells using 100 ppm PEO solution in a 15 mm long rectangular microchannel. The sample flow rate is fixed at 5 µL/min, while sheath fluid has five different flow rates: 10, 20, 30, 40, and 50 µL/min. The flow rate ratio $\alpha$ are 2, 4, 6, 8 and 10, respectively. The Re are 8.3, 13.84, 19.37, 24.91, and 30.44, respectively, and Wi are 3.08, 5.13, 7.18, 9.23, and 11.28, respectively. (a) Experimental images for *B. subtilis* cells with various lengths at the expansion region for five different sheath flow rates. The black dashed lines represent channel centrelines. The scale bar represents 50 µm. (b) Plots of average normalized lateral positions for four groups of *B. subtilis* cells with different lengths: 1-5 μm (blue), 5-10 μm (green), 10-20 μm (purple), and >20 μm (pink). The error bars indicate the standard deviation obtained from at least 100 measurements.


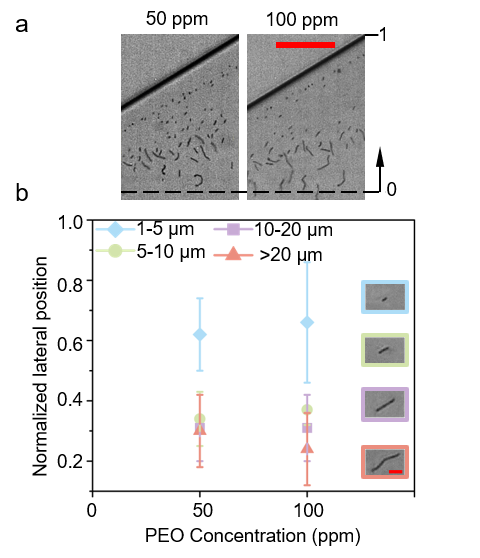


Figure S7 Comparison of the separation of *B. subtilis* cells in a 15 mm long rectangular microchannel at two different PEO concentrations: 50 and 100 ppm. The flow rates of sample and sheath are 5 µL/min and 40 µL/min, respectively. The Re are 25.41 and 24.91, respectively, and Wi are 5.85 and 9.23, respectively. (a) Experimental images for *B. subtilis* cells with various lengths at the expansion region. The black dashed lines represent channel centrelines. The scale bar represents 50 µm. (b) Plots of average normalized lateral positions for four groups of *B. subtilis* cells with different lengths: 1-5 μm (blue), 5-10 μm (green), 10-20 μm (purple), and >20 μm (pink). The error bars indicate the standard deviation obtained from at least 100 measurements. The scale bar represents 10 µm.


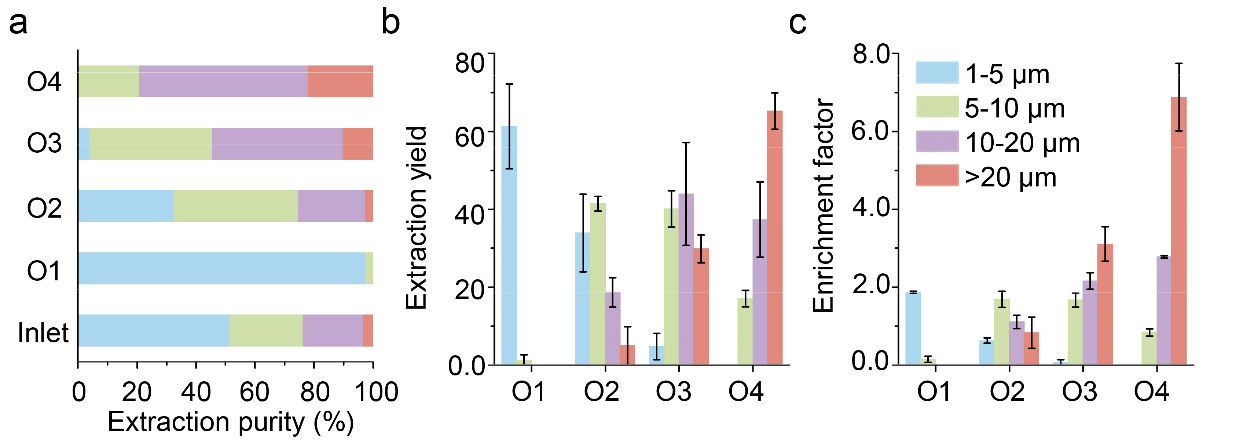


Figure S8 Separation and enrichment of four groups of B. subtilis cells with different lengths at the outlets using 100 ppm PEO solution in a 15 mm long rectangular microchannel. The flow rates of sample and sheath fluids are 5 µL/min and 40 µL/min, respectively. (a) A comparison of EP for four groups of B. subtilis cells: 1-5 μm (blue), 5-10 μm (green), 10-20 μm (purple) and >20 μm (pink) at the inlet and each outlet. (b, c) Bar graphs of (b) EY and (c) EF for the four groups of B. subtilis cells with different lengths for each outlet. The error bars represent the standard deviation of three measurements.


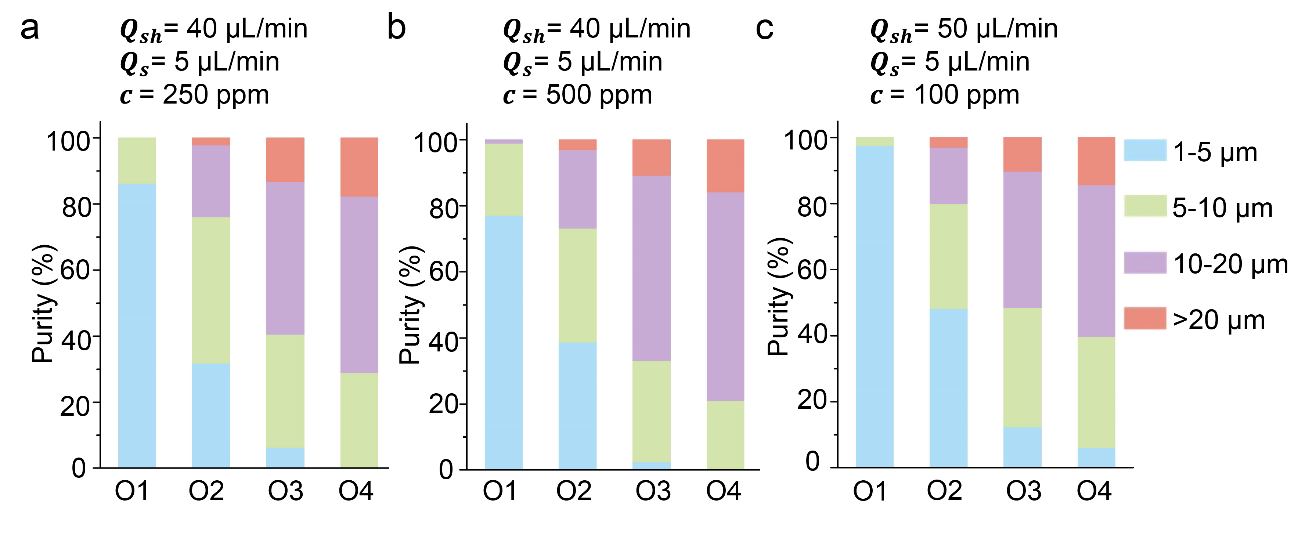


Figure S9 Comparison of EP for four groups of *B. subtilis* cells: 0-5 μm (blue), 5-10 μm (green), 10-20 μm (purple) and >20 μm (pink), at the outlets under different fluid conditions in a 15 mm long rectangular microchannel.

**Supplementary Movies**

**Movie S-1**

Length-based separation of *B. subtilis* at an expansion region.

**Movie S-2**

Short *B. subtilis* cells exit from Outlet 1.

**Movie S-3**

*B. subtilis* cells with different lengths exit from different outlets.
